# Novel Multiplexed HIV/Simian Immunodeficiency Virus Antibody Detection Assay

**DOI:** 10.3201/eid1712.110783

**Published:** 2011-12

**Authors:** Steve Ahuka-Mundeke, Ahidjo Ayouba, Placide Mbala-Kingebeni, Florian Liegeois, Amandine Esteban, Octavie Lunguya-Metila, Didace Demba, Guy Bilulu, Valentin Mbenzo-Abokome, Bila-Isia Inogwabini, Jean-Jacques Muyembe-Tamfum, Eric Delaporte, Martine Peeters

**Affiliations:** University of Montpellier, Montpellier, France (S. Ahuka-Mundeke, A. Ayouba, F. Liegeois, A. Esteban, E. Delaporte, M. Peeters);; Institut National de Recherche Biomédicales, Kinshasa, Democratic Republic of Congo (S. Ahuka-Mundeke, P. Mbala-Kingebeni, O. Lunguya-Metila, J.-J. Muyembe-Tamfum);; Cliniques Universitaires de Kinshasa, Kinshasa (S. Ahuka-Mundeke, P. Mbala-Kingebeni, O. Lunguya-Metila, J.-J. Muyembe-Tamfum);; Zone de Santé de Kole, Sankuru, Kasai Oriental, Democratic Republic of Congo (D. Demba, G. Bilulu);; World Wildlife Fund For Nature, Kinshasa (V. Mbenzo-Abokome, B-I. Inogwabini)

**Keywords:** HIV, simian immunodeficiency virus, SIV, viruses, Democratic Republic of the Congo, Africa, nonhuman primates, bushmeat, multiplexed antibody detection assay

## Abstract

Like most emerging infectious disease viruses, HIV is also of zoonotic origin. To assess the risk for cross-species transmission of simian immunodeficiency viruses (SIVs) from nonhuman primates to humans in the Democratic Republic of Congo, we collected 330 samples derived from nonhuman primate bushmeat at 3 remote forest sites. SIV prevalences were estimated by using a novel high-throughput assay that included 34 HIV and SIV antigens in a single well. Overall, 19% of nonhuman primate bushmeat was infected with SIVs, and new SIV lineages were identified. Highest SIV prevalences were seen in red-tailed guenons (25%) and Tshuapa red colobus monkeys (24%), representing the most common hunted primate species, thus increasing the likelihood for cross-species transmission. Additional studies are needed to determine whether other SIVs crossed the species barrier. With the newly developed assay, large-scale screening against many antigens is now easier and faster.

## Abstract 2

This assay identified new simian immunodeficiency viruses in primate bushmeat.

Like many emerging infectious disease viruses, HIV is also of zoonotic origin (*1*). The closest relatives of HIV-1 are simian immunodeficiency viruses (SIVs), specifically SIVcpz and SIVgor in chimpanzees (*Pan troglodytes troglodytes*) and gorillas (*Gorilla gorilla*), respectively, from west-central Africa (*2**,**3*). SIVsmm in sooty mangabeys (*Cercocebus atys*) from west Africa are the closest relatives of HIV-2 (*4**,**5*). SIVs from mangabeys, gorillas, and chimpanzees crossed the species barrier >12 times (*1**,**6*). Exposure to blood, secretions, or tissues from infected primates through hunting and butchering of bushmeat represents the most plausible source for human infection.

Humans are still hunting and butchering a wide diversity of primate species and the possibility of additional cross-species transfers of viruses has to be considered (*7**,**8*). Recent reports showed ongoing transmission of simian retroviruses to humans in central Africa, i.e., a wide variety of simian foamy viruses and new human T-lymphotropic virus variants, closely related to viruses in co-habiting nonhuman primates, have been observed in humans who report primate hunting and butchering (*9**–**12*). The description in 2009 of HIV-1 group P, closely related to SIVgor, in a patient from Cameroon living in France, shows also that our knowledge on HIV diversity and possible cross-species transmissions is still incomplete and illustrates how rapidly new viruses can spread today to other continents (*6*).

Given the potential pathogenicity of these lentiviruses, as illustrated by the actual HIV-1 group M pandemic that resulted from a single cross-species transmission, it is necessary to estimate to what extent humans are exposed to SIVs and whether other viruses crossed the species-barrier. SIV infection has already been identified in >40 nonhuman primate (NHP) species from Africa but our knowledge on prevalence and geographic distribution remains limited; few large-scale studies on retroviral infections in wild primate populations have been conducted (*13*). SIV prevalences can vary among species and within species according to geographic areas (*2**,**14**,**15*), and exposure to infected primates and subsequent risk for cross-species transmission can thus differ across Africa.

SIV infections were initially identified on the basis of cross-reactivity with HIV antigens (*8*), but to increase sensitivity, SIV lineage-specific ELISAs have been developed. These assays must be regularly updated when new SIV lineages are discovered (*14**–**17*). Therefore, they become time-consuming and bench work–consuming, use relatively large volumes of scarce biological material, and are not adapted for large-scale surveillance studies. We adapted the Multiple Analyte Profiling technology (xMAP; Luminex Inc., Austin, TX, USA), which is a flow cytometry–based system (*18*), for simultaneous antibody detection against 34 peptides representing the actual known HIV/SIV diversity. This new assay was used to study SIV infection in primate bushmeat in the Democratic Republic of Congo (DRC), home to a wide diversity of primate species.

## Materials and Methods

### NHP Samples

**Figure 1:**
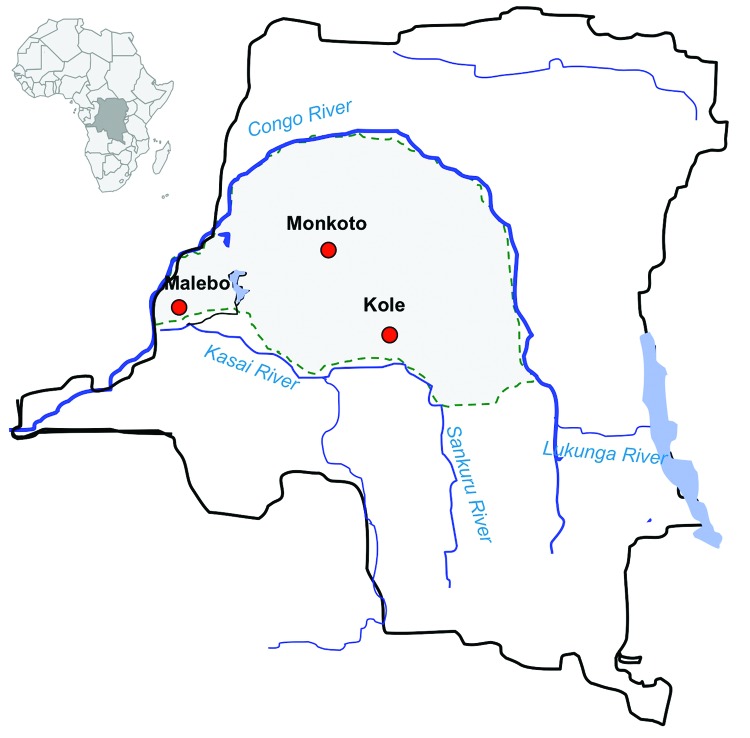
Sites in the Democratic Republic of Congo where dried blood spots of nonhuman primates were collected (red circles).

For the validation of the HIV/SIV xMAP assay, we used 142 well-characterized samples from our NHP reference panel in which SIV infection was either confirmed or ruled out by highly sensitive PCR approaches, and for which sufficient plasma was available (*14**,**15*). The panel included 93 SIV-negative samples from 8 species and 49 SIV-positive samples from 9 species (Table 1). For SIV prevalence studies, 330 samples were collected during May 2009–2010 as dried blood spots (DBSs) around 3 rural cities in DRC (Figure 1). Whole blood, collected from primate bushmeat, was spotted onto a filter 903 FTA card (Whatman Plc, Kent, UK). After air-drying at ambient temperature, DBSs were stored into individual envelopes at ambient temperature. Animals died 6–78 hours before sampling. All NHP samples were obtained with approval from the Ministry of Environment and Health and the National Ethics Committee. Similar to our previous studies, bushmeat samples were obtained through a strategy specifically designed not to increase demand (*8**,**15*).

**Table 1:** Panel of reference serum samples from SIV-infected and -noninfected nonhuman primate species*

Species (common name)	SIV lineage	No. samples	
		SIV+	SIV–
*Pan troglodytes troglodytes* (west- central chimpanzee)	SIVcpz*Ptt*	2	1
*Pan troglodytes schweinfurthii* (eastern chimpanzee)	SIVcpz*Pts*	1	NF
*Cercopithecus nictitans* (greater spot-nosed monkey)	SIVgsn	6	45
*Cercopithecus cephus* (mustached monkey)	SIVmus	7	30
*Cercopithecus mona* (Mona monkey)	SIVmon	NF	1
*Miopithecus ogouensis* (northern talapoin)	SIVtal	1	6
*Cercopithecus neglectus* (De Brazza monkey)	SIVdeb	7	1
*Cercocebus torquatus* (red-capped mangabey)	SIVrcm	4	NF
*Chlorocebus tantalus* (African green monkey)	SIVagm	1	NF
*Mandrillus sphinx* (mandrill)	SIVmnd-2	10	2
*Colobus guereza* (mantled guereza)	SIVcol	10	7
Total		49	93

*NF, none found.

### Screening for Cross-Reactive HIV/SIV Antibodies

All DBS samples were screened with the new HIV/SIV multiplex microbead immunoassay technology, i.e., xMAP. Similarly as for the SIV ELISAs, we used peptides covering the immunodominant region of the gp41 transmembrane and V3-loop region from all major SIV/HIV lineages known at the time we conducted this study (Table 2). To avoid interpeptide and intrapeptide cross-linking, the 2 cysteins of the gp41 peptides were cyclicized during synthesis. For SIVcol, gp41 peptides could not be synthesized because of their low solubility; the V3-loop peptide was used to identify corresponding antibodies (*14**,**15*). Peptides were covalently coupled on carboxyl-functionalized fluorescent polystyrene beads (Luminex Inc.) by using the Bio-Plex Amine Coupling Kit (Bio-Rad Laboratories, Marnes-la-Coquette, France) according to the manufacturer’s instructions. Unreacted sites were blocked with blocking buffer from the Amine Coupling Kit (Bio-Rad Laboratories). Peptide-coupled microsphere preparations were counted by using a hemocytometer and stored in the dark at 4°C. Before use, peptide-coupled beads were vortexed (30 s), sonicated (30 s), and diluted to 4,000 beads/µL. Dilution and washing buffer consisted of phosphate-buffered saline (PBS) containing 0.75 mol/L NaCl, 1% (wt/vol) bovine serum albumin (Sigma Aldrich, St. Quentin Fallavier, France), 5% (vol/vol) fetal bovine serum (Gibco-Invitrogen, Cergy Pontoise, France), and 0.05% (vol/vol) Tween-20 (Sigma-Aldrich).

**Table 2:** Amino acid sequences of the 34 SIV/HIV peptides used to develop the xMAP gp41 and V3-loop multiplex Luminex assay

Peptide	SIV lineage	gp41 peptide sequences	V3-loop peptide sequences
HIV-1 M	HIV1/SIVcpz/gor	LAVERYLKDQQLLGIWGCSGKLIC	NNTRKSVRIGPGQAFYATGDIIGDIRQAYC
HIV-1 O	HIV1/SIVcpz/gor	LALGTLIQNQQLLNLWGCKGKLIC	NLTVQEIKIGPMAWYSMGLAAGNGSRAYC
HIV-1 N	HIV1/SIVcpz/gor	LAIGRYLRDQQILSLWGCSGKTIC	NNTGGQVGIGPAMTFYNIGKIVGDIRKAYC
SIVcpz*Pts*	HIV1/SIVcpz/gor	LAVEKYLRDQQLLSLWGCADKVTC	NRTVRNLQIGPGMTFYNVEIATGDTRKAFC
SIVcpz*Ptt*	HIV1/SIVcpz/gor	LAVERYLQDQQILGLWGCSGKAVC	NNTRGEVQIGPGMTFYNIENVVGDTRSAYC
SIVgor	HIV1/SIVcpz/gor	LAIETYLRDQQLLGLWGCTGKLIC	NNTRGQIQIGPMTIYNSERIIGNTRKAYC
HIV-2/SIVsmm	HIV-2/SIVsmm	TAIEKYLKDQAQLNSWGCAFRQVC	GNKTVVPITLMSGLVFMSQPINKRPRQAWC
SIVrcm	SIVrcm	TAIEKYLADQSLLNTFGCAWRQVC	SNRTVKGISLAIGVFISLRVEKRPKGAWC
SIVagm	SIVagm	TALEKYLEDQARLNAWGCAWKQVC	GNKTVLPVTIMAGLVFHSQKYNTLLRQAWC
SIVgsn	SIVgsn/mus/mon†	SSLEKYLRDQTILQAWGCANRPIC	GNKTIRNLQIGAGMTFYSQVIVGGNTRKAYC
SIVmus	SIVgsn/mus/mon†	TALEKFVKDQAILNLWGCANRQIC	†
SIVmon	SIVgsn/mus/mon†	TAVEKFIKDQTLLNAWGCANKAVC	†
SIVdeb	SIVdeb	TAIEKYLKDQAKLNEWGCAFKQIC	GNKTYRAVHMATGLSFYTTFIPRLRIKRAHC
SIVtal	SIVtal‡	TALEKYLEDQAKLNSWGCAWKQIC	*RTIKDLQIAAGLMFHSQIIAGKDLKRAY*
SIVsyk	SIVsyk	TALETYLRDQAIMSNWGCAFKQIC	GNESIKNIQLAAGYFLPVIQGKLKTGRDAKRAFC
SIVlho	SIVlho/sun	TAIEEYLKDQALLASWGCQWKQVC	GNRSEVSTISSTGLLFYYGLEHGSRLRLAQC
SIVmnd-2	SIVmnd	TALEDYVADQSRLAVWGCSFSQVC	GNRSVVSTPSATGLLFYHLGPGKNLKKGMC
SIVwrc	SIVwrc	SAIEGFLEDQLKLKQWGCELTQVC	GNRSVVSVNSASGLIYYAGLEPHRNIRKGLC
SIVcol	SIVcol‡	*ATIEGYLEEQAKLASIGCANMQIC*	GNSSHRNLNTANGAKFYYELIPYSKGIYGRC

*SIV, simian immunodeficiency virus. †V3 amino acid sequences of SIVgsn, SIVmus, and SIVmon were identical, and only the SIVgsn above the V3-loop peptide was synthesized. ‡Synthesis of SIVcol gp41 and SIVtal V3-loop peptides was unsuccessful.

Assays were performed in 96 well flat-bottomed filter plates (Millipore, Tullagreen, Ireland). Plates were prewet with 100 µL assay buffer; 50 μL of bead mixture was subsequently added to each well. Liquid was aspirated with a vacuum manifold and wells were washed with 100 μL of assay buffer. Wells were then incubated with 100 μL plasma (diluted 1:200) for 60 min at room temperature in the dark on a plate shaker (300 rpm/min). For DBSs, 100 μL of eluate, obtained after overnight incubation of two 6-mm disks in 1 mL of hypertonic PBS, was added to peptide-coated beads. After washing, plates were incubated with 50 μL/well of 4 μg/mL biotinylated mouse antihuman IgG (BD Pharmingen, Le Pont de Claix, France) for 30 min at room temperature in the dark under continuous shaking. After washing, wells were incubated with 50 μL of 1 μg/mL streptavidin-R-phycoerythrin conjugate/well (Invitrogen/Molecular Probes, Cergy Pontoise, France) for 10 min in the dark while shaking. After 2 final washes with reading buffer (PBS containing 1% [wt/vol] bovine serum albumin), beads were resuspended in 125 µL reading buffer/well and analyzed by using BioPlex-200 (Bio-Rad Laboratories).

Data were analyzed by using BioPlex Software Manager version 5.0 (Bio-Rad Laboratories). For each bead set, >100 events were read and results were expressed as median fluorescence intensity (MFI) per 100 beads. The cutoff value was calculated for each peptide as the mean MFI for all antibody-negative reference serum samples plus 5 SD as an adaptation of the strategy defined for ELISA and was set at 200 MFI corresponding to a consensus value for all peptides (*19*). Sensitivity and specificity were calculated for homologous (same species) and heterologous (different species) antibody detection.

DBS samples were also tested for HIV cross-reactive antibodies by using the INNO-LIA HIV Confirmation Test (Innogenetics, Gent, Belgium) as described (*10*). This test configuration includes HIV-1 and HIV-2 recombinant proteins and synthetic peptides that are coated as discrete lines on a nylon strip.

### DNA Extraction and NHP Species Confirmation

Total DNA was extracted from all DBSs by using the Nuclisens MiniMAG Extraction Kit (Biomerieux, Craponne, France) according to the manufacturer’s instructions. Minor changes consisted of increasing the incubation time (2 h) of the viral lysis step to increase DNA release (*20*). Species identification recorded in the field was confirmed on all samples by amplifying a 386-bp mitochondrial DNA fragment of the 12S rRNA gene with primers 12S-L1091 and 12S-H1478 (*21*). PCR products were purified by electrophoresis on a 1% agarose gel and directly sequenced (ABI PRISM Big Dye Terminator Cycle sequencing Ready Reaction Kit with amplitaq FS DNA polymerase) on an automated sequencer (ABI 3130XL, Applied Biosystems, Courtaboeuf, France). Sequences were then assembled by using the software package Lasergene (DNASTAR, Inc, Madison, WI, USA).

### Molecular Characterization and Phylogenetic Analyses of SIVs

PCR analyses were performed on SIV antibody-positive samples by using described conditions with universal HIV/SIV and SIV lineage-specific primers in *pol* or *env* shown in Table 3 (*8**,**15**,**22**–**24*). PCR products were purified by electrophoresis on a 1% agarose gel and directly sequenced as described above. Newly derived SIV nucleotide sequences were aligned with reference sequences of the different HIV/SIV lineages with MEGA4 and ClustalX version 2 (*25*) and minor manual adjustments when necessary. Nucleotide sites that could not be unambiguously aligned were excluded from the analyses. Appropriate models of evolution were selected for each data set by using Topali software (*26*), and maximum-likelihood phylogenies were reconstructed by using PhyML (*27*). The analyses were performed by using discrete gamma distribution and generalized time-reversible model. The starting tree was obtained by using PhyML. One hundred bootstrap replications were performed to assess confidence in topology. New sequences have been deposited in GenBank under accession nos. JN020273–JN020279 and GU989632.

**Table 3:** Primers used to amplify simian immunodeficiency virus from dried blood spot samples

Primer	Sequences, 5′ → 3′*	Region targeted	Estimated amplicon size, bp	References
DR1	TRCAYACAGGRGCWGAYGA	*pol*	800	(*8*,*22*)
DR2	AIADRTCATCCATRTAYTG			
DR4	GGIATWCCICAYCCDGCAGG		200	
DR5	GGIGAYCCYTTCCAYCCYTGHGG			
polis4†	CCAGCNCACAAAGGNATAGGAGG	*pol*	800	(*8*,*22*)
polOR†	ACBACYGCNCCTTCHCCTTTC			
polis2†	TGGCARATRGAYTGYACNCAYNTRGAA		400	
uni2†	CCCCTATTCCTCCCCTTCTTTTAAAA			
polis4†	CCAGCNCACAAAGGNATAGGAGG	*pol*	800	(*8*,*22*)
polOR†	ACBACYGCNCCTTCHCCTTTC			
polis4†	CCAGCNCACAAAGGNATAGGAGG		650	
uni2†	CCCCTATTCCTCCCCTTCTTTTAAAA			
CNMF1	TATCCYTCCYTGTCATCYCTCTTT	*pol*	2,750	(*23*)
POLor2	ACBACWGCTCCTTCWCCTTTCCA			
CNMF2	AATGGAGAATGYTMATAGATTTCAG		2,050	
CNMR	CCCCYATTCCTCCCTTTTTTTTA			
SPBS	GGCGCCCGAACAGGGACTTG	*gag-pol*	2,500	(*23*)
2500P1	CCTCCTATGTTCCCCTATTTCTCTG			
CNM.G1	CGAGGCACTCGGCGATGCTGA		2,200	
2500P2	GGAACTGAGAAGGCTGTGTAAGGC			
2500L1	CTATCCCCAAACGCATCCGC	*env-gag*	2,000	(*23*)
CNM.G1rev	TCAGCATCGCCGAGTGCCTCG			
2500L2	AGAAAAGGGAGGACTGGAAGGGAT		800	
SPBSrev	CAAGTCCCTGTTCGGGCGCC			
CNMenvF1	TGTGTSAAAYTRACHCCNATGTGTGT	*env*	2,480	(*23*)
CNMenvR1	AACATNNCYTCYAGTCCTCYCTTTTYT			
CNMenvF2	TCCTTYAAYCAGACYACAGARTTYAGRGA		2,140	
CNMenvR1	GGGATAGCCANGAATTNTCNCCAT			
wrcpolF1	TAGGGACAGAAAGTATAGTAATHTGG	*pol*	1,100	(*24*)
wrcpolR1	GCCATWGCYAA TGCTGTTTC			
wrcpolF2	AGAGACAGTAAGGAAGGGAAAGCAGG		650	
wrcpolR2	GTTCWATTCCTAACCACCAGCADA			
wrcenvF1	TGGC AGTGGGACAAAAATATAAAC	*env*	750	(*24*)
wrcenvR1	CTGGCAGTCCCTCTTCCA AGTT GT			
wrcenvF2	TGATAGGGMTGGCTCCTGGTGATG		550	
wrcenvR2	AATCCCCATTTYAACCAGTTCCA			

*R = A or G; M = A or C; W = A or T; S = G or C; Y = C or T; B = C, G, or T; H = A, C, or T; and N = A, C, G, or T. †Primers that amplifie simian immunodeficiency virus from dried blood spot samples in this study.

## Results

### Performance of the HIV/SIV Lineage Specific xMAP Assay on a Reference Panel of NHP Samples

Table 4 summarizes the sensitivity and specificity of homologous and heterologous antibody detection of the xMAP assay on the same reference panel that was used for the SIV lineage–specific ELISAs (*17**,**18*). The homologous gp41 peptide was available for 39 samples; 34 (87.2%) reacted with their gp41 peptide counterpart. Similarly, 46 (93.9%) of the 49 samples for which the homologous V3 peptide was available reacted with their V3 peptide counterpart. The combination of homologous gp41 and V3 peptides identified 47 (95.9%) of the 49 SIV-positive samples. All 49 SIV positive samples were identified by combining homologous and heterologous gp41 reactivities, including SIVcol positive samples for which no homologous gp41peptide was available, resulting in 100% sensitivity. The 3 SIVmnd samples that were not detected by the homologous gp41 peptide were all detected with the SIVmnd V3 peptide, and the SIVcpz*Ptt* sample that was not detected by the SIVcpz peptides was reactive with the HIV-1 N gp41 peptide. Each gp41 peptide cross-reacted with >1 sample from a different primate species (data not shown); highest cross-reactivities were for SIVmus (23/48, 47.9%) and SIVsmm (30/48, 62.5%) peptides. Finally, none of the negative serum samples showed positive results with homologous gp41 or V3 peptides. However, 2 (1 *Cercopithecus* *nictitans* and 1 *C. cephus* monkeys) reacted weakly (MFI/cutoff ratio <2) with a single heterologous V3 peptide from SIVagm, resulting in an overall 100% and 97.9% specificity of homologous and heterologous antibody detection, respectively. Given the extraordinary SIV diversity, few false-negative samples were observed, and the combination of all peptides in a single well resulted in 100% sensitivity and 97.5% specificity. Thus, the new assay should enable detection of most SIV infections.

**Table 4:** Sensitivity and specificity of SIV/HIV peptides used in the xMAP assay to detect SIV infection in human and nonhuman primate samples*

Species (common name)	Peptide	Homologous detection								Homologous and heterologous detection				
		SIV+				SIV–				SIV+			SIV–	
		gp41	V3	gp41 + V3		gp41	V3	gp41 + V3		gp41	V3		gp41	V3
*Pan troglodytes troglodytes* (west central chimpanzee)	SIVcpz*Ptt*	1/2	1/2	1/2		0/1	0/1	0/1		3/49	4/49		0/93	0/93
*Pan troglodytes schweinfurthii* (eastern chimpanzee)	SIVcpz*Pts*	1/1	1/1	−/−		−/−	−/−	−/−		8/49	13/49		0/93	0/93
*Gorilla gorilla gorilla* (western lowland gorilla)	SIVgor	−/−	−/−	−/−		−/−	−/−	−/−		1/49	0/49		0/93	0/93
*Cercopithecus nictitans* (greater spot-nosed monkey)	SIVgsn	5/6	6/6	6/6		0/45	0/45	0/45		14/49	15/49		0/93	0/93
*Cercopithecus cephus* (mustached monkey)	SIVmus	7/7	7/7	7/7		0/30	0/30	0/30		23/49	15/49		0/93	0/93
*Cercopithecus mona* (Mona monkey)	SIVmon	−/−	−/−	−/−		0/1	0/1	0/1		12/49	15/49		0/93	0/93
*Miopithecus ogouensis* (northern talapoin)	SIVtal	1/1	−/−	−/−		0/6	0/6	0/6		6/49	−/−		0/93	0/93
*Cercopithecus neglectus* (De Brazza monkey)	SIVdeb	7/7	6/7	7/7		0/1	0/1	0/1		19/49	6/49		0/93	0/93
*Cercopithecus albogulari*s (Sykes’ monkey)	SIVsyk	−/−	−/−	−/−		−/−	−/−	−/−		11/49	1/49		0/93	0/93
*Cercocebus atys* (sooty mangabey)	SIVsmm	−/−	−/−	−/−		−/−	−/−	−/−		30/49	1/49		0/93	0/93
*Cercocebus torquatus* (red-capped mangabey)	SIVrcm	4/4	4/4	4/4		−/−	−/−	−/−		7/49	4/49		0/93	0/93
*Chlorocebus tantalus* (African green monkey)	SIVagm	1/1	1/1	1/1		−/−	−/−	−/−		5/49	3/49		0/93	2/93
*Mandrillus sphinx* (mandrill)	SIVmnd-2	7/10	10/10	10/10		0/2	0/2	0/2		13/49	11/49		0/93	0/93
*Cercopithecus lhoesti* (L'Hoest's monkey)	SIVlho	−/−	−/−	−/−		−/−	−/−	−/−		6/49	6/49		0/93	0/93
*Procolobus badius* (western red colobus)	SIVwrc	−/−	−/−	−/−		−/−	−/−	−/−		1/49	6/49		0/93	0/93
*Colobus guereza* (mantled guereza)	SIVcol	−/−	9/10	9/10		−/−	0/7	0/7		−/−	9/49		0/93	0/93
*Homo sapiens* (human)	HIV-1M	−/−	−/−	−/−		−/−	−/−	−/−		4/49	1/49		0/93	0/93
	HIV-1-O	−/−	−/−	−/−		−/−	−/−	−/−		1/49	0/49		0/93	0/93
	HIV-1N	−/−	−/−	−/−		−/−	−/−	−/−		9/49	1/49		0/93	0/93
Total		34/39	46/49	47/49		0/87	0/87	0/87		49/49	47/49		0/93	2//93
Sensitivity		87.2%	93.9%	95.9%		NA	NA	NA		100%	95.5%		NA	NA
Specificity		NA	NA	NA		100%	100%	100%		NA	NA		100%	97.9%

*SIV, simian immunodeficiency virus; −/−, not calculated because homologous serum and/or peptide not available; NA, not applicable.

### NHP Species Collected as Bushmeat at the Different Localities in DRC

DBS samples were obtained from 330 NHPs in 3 sites, but most (258/330, 78.2%) were collected around Kole (Figure 1). Species were identified in the field by pictographs and confirmed by sequence analysis of the 12S rRNA gene. This analysis identified 7 species: 147 yellow-nosed red-tailed guenons (*C. ascanius whitesidei*), 79 Tshuapa red colobus monkeys (*Piliocolobus tholloni)*, 33 Wolf’s monkeys (*C. mona wolfi)*, 33 black mangabeys (*Lophocebus atterrimus atterrimus)*, 25 Angolan pied colobus (*Colobus angolensis angolensis)*, 10 De Brazza monkeys (*C. neglectus),* and 3 Allen swamp monkeys (*Allenopithecus* *negroviridis*) (*28*). Four of the 7 species or subspecies are only present in DRC, i.e., red-tailed guenons, tshuapa red colobus, black mangabeys, and Wolf’s monkeys (*28*).

### Prevalence and Genetic Diversity of SIVs in DRC

Because 4 of the 7 NHP species or subspecies are only present in DRC, they are most likely infected with SIVs that have not been documented, and antibody detection will thus depend on extent of cross-reactivity with antigens of known HIV/SIV lineages. Therefore, we screened all samples with the new SIV/HIV xMAP assay and with the HIV line immune assay (INNO-LIA) confirmation assay, which we previously used to detect a large diversity of SIV infections (*8*). Table 5 shows the number of SIV-positive and indeterminate samples per species. SIV infection was documented in 6 species and the overall prevalence was 19% (64/330) ranging from 0% to 25% per species. Highest SIV prevalences were seen in red-tailed guenons (25%) and Tshuapa red colobus (24%), which represent 70% (226/330) of the primate bushmeat collected in this study. We also observed HIV/SIV cross-reactive antibodies in De Brazza monkeys (20%), Wolf’s monkeys (12 %), black mangabeys (3%), and Angola pied colobus (4%). In addition, 3% (10/330) of the samples were considered as indeterminate for SIV. Notably, all samples from Tsuapa red colobus were only reactive in the xMAP assay and showed strong cross-reactivity with SIVwrc antigens from western red colobus (*Piliocolobus badius*), illustrating clearly the need for including a wide variety of SIV antigens to uncover new SIV lineages.

**Table 5:** Number and percentage of SIV antibody–positive samples per species and number of samples confirmed by PCR and sequence analysis per species and per site*

Species (common name)	Antibody detection, no. (%)														PCR, no. pos/no. tested
	Kole				Malebo				Monkoto†			Total			
	Tested	Pos	Ind		Tested	Pos	Ind		Tested	Pos		Tested	Pos	Ind	
*Allopithecus negroviridis* (Allen's swamp monkey)	1	0	0		NA	NA	NA		2	0		3	0	0	Not done
*Colobus angolensis* (Angolan colobus)	21	1 (5.0)	0		NA	NA	NA		4	1 (25.0)		25	1 (4.0)	0	0/1
*Cercopithecus ascanius* (red-tailed monkey)	94	23 (24.4)	4 (4.2)		40	12 (30.0)	3 (7.5)		13	3 (23.0)		147	37 (25.0)	7 (4.7)	4/37
*Cercopithecus neglectus* (De Brazza monkey)	7	2 (28.0)	0		2	0	0		1	0		10	2 (20)	0	1/2
*Cercopithecus wolfi* (Wolf's monkey)	27	5 (19.0)	3 (11.0)		1	0	0		5	3 (60.0)		33	4 (12.1)	3 (9.1)	1/4
*Lophocebus atterimus* (black mangabey)	30	2 (6.0)	0		NA	NA	NA		3	0		33	1 (3.0)	0	0/1
*Piliocolobus tholloni* (Tshuapa red colobus)	78	19 (24.0)	0		NA	NA	NA		1	0		79	19 (24.1)	0	2/19
Total	258	52 (20.0)	7 (3.0)		43	12 (27.0)	3 (7.0)		29	7 (21.0)		330	64 (19.0)	10 (3.0)	8/64

*SIV, simian immunodeficiency virus; pos, positive; ind, indeterminate; NA, not applicable (samples not available). Samples were scored as SIV positive when both assays (xMAP and HIV INNO-LIA) were clearly positive or positive in 1 assay (MFI/cutoff >2 or reactivity with at least 1 HIV antigen with a band intensity equal to or greater than the assay cutoff). Samples were scored indeterminate (ind) when reactive in HIV INNO-LIA with at least one HIV antigen with a band intensity equal to or greater than the assay cut-off and weakly positive (MFI/cut-off ratio between 1 and 2) in the other assay or when both assays were indeterminate; samples were considered as negative when both test results were negative. †No samples from Monkoto were indeterminate.

### Genetic Diversity of SIVS in DRC

**Figure 2:**
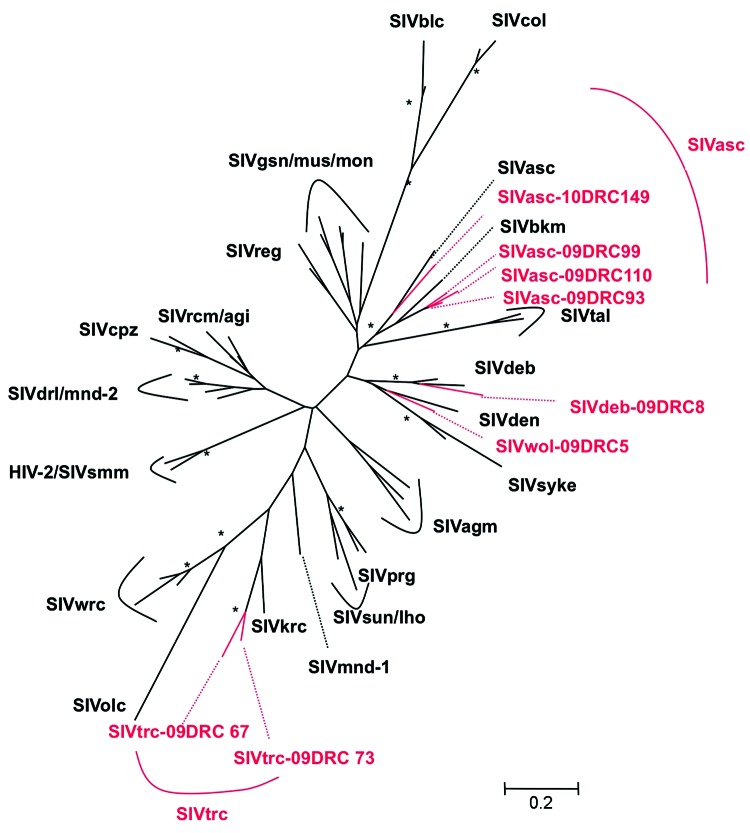
Phylogenetic relationships of the newly derived simian immunodeficiency virus (SIV) sequences in *pol* to representatives of the other SIV lineages. Newly identified strains in this study are in red and reference strains are in black. Unrooted trees were inferred from 350-bp nucleotides. Analyses were performed by using discrete gamma distribution and a generalized time reversible model. The starting tree was obtained by using phyML (*27*). One hundred bootstrap replications were performed to assess confidence in topology. Numbers at nodes are from 100 bootstrap replicates; only those >90% are shown with an asterisk. Scale bar represents nucleotide replacements per site.

To confirm SIV infection and document SIV diversity, all SIV-positive and indeterminate samples were subjected to PCR amplification. Although DNA integrity was sufficient to confirm the primate species in all DBSs, proviral SIV DNA could only be amplified in *pol* (400 bp) for 8 samples, most likely because of DNA degradation related to long and suboptimal storage at ambient temperature in the field and the fact that animals died several days before sampling. SIV infection was confirmed in 4 red-tailed guenons, 1 Wolf’s monkey, 1 De Brazza monkey, and 2 Tshuapa red colobus. Phylogenetic tree analysis shows the presence of new SIV lineages in Wolf‘s monkeys and Tshuapa red colobus (Figure 2). SIVwol is close to SIVden obtained from Dent’s monkeys (*C. mona denti*), which are found in eastern DRC but without overlapping habitats with Wolf’s monkeys in central DRC (*28*). SIVtrc from Tshuapa red colobus forms a separate lineage although related to SIVkrc from Kibale red colobus in eastern Africa (*29*). SIVasc from red-tailed guenons forms a species-specific lineage with SIVasc described in a capitive animal, but a high genetic diversity is seen (*30*). The reported *pol* sequence from a captive black mangabey housed in the zoo in Kinshasa, also falls within the SIVasc radiation (*31*). Finally, SIVdeb clustered within the species-specific SIVdeb lineage observed for De Brazza monkeys across central Africa (*32*).

## Discussion

In this study, we used a novel high throughput immune assay that included 34 HIV and SIV antigens in a single well to evaluate prevalence and genetic diversity of SIVs from NHPs at the primate/human interface in DRC. Overall, we showed that ≈20% of NHP bushmeat is infected with SIVs and identified new SIV lineages. Highest SIV prevalences were seen among the most commonly hunted primate species.

Although SIV lineage-specific ELISAs were highly sensitive and specific (*14**–**17*), with the increasing number of new SIV lineages and the high genetic diversity within SIV lineages, a large number of antigens must be included. Using a large set of SIV antigens is necessary, especially when new species are tested for which no SIVs have been reported and when antibody detection is based on cross-reactivity with antigens from heterologous SIV/HIV lineages. To reduce increasing workload and volumes of scarce biological material, we adapted xMAP technology to enable a single sample to be tested simultaneously for multiple peptides (*18*). We used the same gp41 and V3-loop peptides as in the SIV lineage-specific ELISAs and the same reference panel of NHP samples to validate the assay (*14**,**15*). We updated the assay with antigens of SIVgor from gorillas (*3*) and HIV-1 groups M, N, and O. The homologous reactivity for some gp41 peptides, especially SIVmnd, was lower in the xMAP assay (95.9%) compared with that of ELISAs (97.5%) (*15*). However, these samples were also only weakly reactive in the gp41 ELISA, suggesting that all antigens in a single well could slightly reduce sensitivity when mismatches are present in corresponding gp41 sequences. The combination of 34 peptides in a single well detected SIV infection in the reference panel with 100% sensitivity and 97.9% specificity and reduced workload and volumes of biological material. The need for including a wide diversity of SIV antigens was clearly illustrated by identification of SIVtrc in Tshuapa red colobus samples, which showed negative results with the INNO-LIA HIV confirmatory assay.

No extensive studies have been conducted on SIV infection in monkeys from DRC, which harbors many species because of the geographic barriers constituted by the Congo, Ubangui, and Kasai Rivers (*28*). Overall, ≈20% of primate bushmeat was SIV infected, and as observed in previous studies, prevalences varied per species (*17**,**18*). We confirmed SIV infection in De Brazza monkeys and red-tailed guenons (*14**,**15**,**30*) and identified new SIV lineages in Wolf’s monkeys (SIVwol) and Tshuapa red colobus (SIVtrc). De Brazza monkeys seem widely infected with SIVdeb across central Africa: 20% in DRC and 40% in Cameroon (*15*). A high genetic diversity is seen in the SIVasc lineage, and this lineage also includes the previously reported SIVbkm sequence from a captive black mangabey from the zoo in Kinshasa, DRC (*31*). Attempts to amplify an SIV in a wild black mangabey in our study were unsuccesful, and more studies are needed to clarify whether the initial SIVbkm infection is caused by contamination from a red-tailed monkey in captivity or in the wild because black mangabeys share habitats with *Cercopithecus* species. Finally, only full-length genome sequences will enable understanding of the evolutionary history of the new SIVwol and SIVtrc viruses.

In addition to many other factors, risk for cross-species transmissions most likely depends on frequency of human contacts with infected primates and on prevalences in frequently hunted species (*33*). For example, SIVcpz*Ptt* and SIVsmm prevalences are highest (30% and 50%, respectively) in areas in west-central and western Africa where precursors of HIV-1 M (M and N) and HIV-2 (A and B) have been identified in chimpanzees and mangabeys, respectively (*2**,**34*). In contrast to our study on SIV prevalences in primate bushmeat in Cameroon, in which we showed <3% seroprevalence (*15*), we observed in DRC high rates of SIV infection (19%) and highest prevalences in the 2 most commonly hunted species. This observation shows clearly that exposure to SIV differs across Africa and that the likelihood for SIVs to cross the species barrier could be higher in DRC than in Cameroon. In addition, contemporary simian foamy virus infections from Angolan pied colobus and red- tailed guenons have been identified in persons living around an area where we collected samples from primate bushmeat, thus confirming ongoing cross-species transmission with simian retroviruses (*35*).

Given the enormous size of the country and absence of road infrastructure, persons in DRC rely on bushmeat for subsistence in many areas. Among ≈70 million inhabitants, 60% live in rural areas (*36*). DRC is recognized as the area where the HIV-1 group M epidemic originated (*37*) but exact conditions associated with epidemic spread of HIV in this part of the world are still incomplete. The internal and regional armed conflicts in DRC that started in 1997 have led to profound socioeconomic changes and internal displacement of human populations (*38*). The long period of civil unrest that followed the outbreak of those conflicts damaged the health care system, and today 70% of the population has little or no access to health care, including HIV/AIDS services (*36*). New epidemic outbreaks, especially with diseases having a long incubation period, can go unrecognized for a long period. In addition, these SIV strains are not recognized by commercial HIV-1/HIV-2 screening assays. A multiplying factor is the presence of logging and mining industries in remote areas that provide favorable conditions for increased human contact with primates and exchanges between urban and rural settlements. High HIV prevalence was reported around logging industries and in displaced population groups (*39**,**40*), which could lead to recombinants between HIVs and SIVs and enable more efficient adaptation and replication in the new host in addition to rapid and further spread to other geographic areas.

Prevalences and high exposure are among the factors that most likely play a role in the transmission of certain SIVs and other simian retroviruses to humans, but viral and host factors also play a role in establishing efficient infection and disease. Given the ongoing contacts between infected NHP and African populations through hunting and butchering, it is likely that SIV cross-transmissions are still occurring. Viruses can remain unrecognized because of a weak health infrastructure and because they are not detected by commercial HIV screening assays. With increasing travel, new viruses can reach other areas rapidly, which have favorable conditions for epidemic spread. It will be necessary to determine whether other SIVs crossed the species barrier, especially in human populations exposed to highly infected primates and in populations with risk behavior that is associated with high epidemic spread. With the new assay that we developed in this study, large-scale screening against a wide variety of antigens is now easier and faster.
